# 2-week prevalence and associated factors of fever, diarrhea, and coexisting fever and diarrhea among children aged 6–23 months in rural Hunan Province

**DOI:** 10.1038/s41598-024-64967-9

**Published:** 2024-06-15

**Authors:** Huixia Li, Juan Xiao, Minghui Liao, Lijia Wan, Qun Huang, Binbin Feng, Xianglian Peng, Ying Tu, Guangwen Huang

**Affiliations:** 1 Department of Child Health Care, Hunan Provincial Maternal and Child Health Care Hospital, Changsha, 410008 Hunan Province China; 2 Hunan Provincial Clinical Research Center for Newborn Diseases Of Maternal Origins, Changsha, 410008 Hunan Province China; 3 Department of Maternal and Children Health, Xiangya School of Public Health, Central South University, Changsha, 410008 Hunan Province China

**Keywords:** Health care, Risk factors

## Abstract

Fever and diarrhea are key causes of malnutrition, growth and development disorders, and death among children. At present, most studies on the associated factors of fever and diarrhea in children are concentrated in African and South Asian countries, but relevant research in China is very limited. This study was aimed to analyze the two-week prevalence of fever, diarrhea, and coexisting fever and diarrhea among children aged 6–23 months in rural areas of Hunan Province and to explore the associated factors. The survey data of the Nutrition Improvement Program for Children in Poor Areas (NIPCPA) from 2016 to 2023 was used here. NIPCPA is a cross-sectional survey completed annually in Hunan to collect children’s nutrition and health indicators. The two-week prevalence rates of fever, diarrhea, and coexisting fever and diarrhea among children aged 6–23 months were 12.2% (2066/16,985), 9.6% (1634/16,985), and 3.2% (542/16,985), respectively. Multivariate logistic regression analysis showed the risks of fever, diarrhea, and coexisting fever and diarrhea were higher among younger children. The high educational level of caregivers, effective consumption of Yingyangbao (a complementary food supplement containing iron, zinc, calcium, vitamins A, D, B1, B2, B12, folic acid, and other micronutrients), and complementary feeding meeting minimum dietary diversity and meeting minimum acceptable diet were protective factors against fever in children, with adjusted odds ratios (aORs) of 0.87 (95%CI: 0.78–0.98), 0.78 (0.69–0.87), 0.73 (0.65–0.82), and 0.74 (0.66–0.84), respectively. Effective consumption of Yingyangbao, and complementary feeding meeting the minimum dietary diversity and meeting minimum acceptable diet were protective factors against diarrhea in children, with aORs of 0.72 (95%CI: 0.63–0.83), 0.79 (0.70–0.91), and 0.80 (0.70–0.92), respectively. Effective consumption of Yingyangbao, and complementary feeding meeting the minimum dietary diversity and meeting minimum acceptable diet were protective factors against coexisting fever and diarrhea among children, with aORs of 0.53 (95%CI: 0.43–0.66), 0.71 (0.58–0.89), and 0.70 (0.56–0.88), respectively. Fever, diarrhea, and the coexisting fever and diarrhea affect one in eight, one in ten, and one in thirty children respectively in rural areas of Hunan. Effective interventions should be actively taken, such as improving the education level of caregivers, enhancing their scientific feeding skills for children, and promoting children’s compliance with Yingyangbao consumption, to further reduce the prevalence of fever and diarrhea in children.

## Introduction

Fever refers to an increase in body temperature beyond the normal range and by more than 1 °C above its basal level, under the action of pyrogen or due to various reasons causing dysfunction in the body temperature regulation center^1^. According to World Health Organization (WHO), children with rectal temperature exceeding 37.5 °C or axillary temperature exceeding 37.0 ℃ are considered to have fever^2^. Diarrhea is a gastrointestinal syndrome characterized by increased stool frequency and changes in stool properties^1^. WHO defines diarrhea as the passage of three or more loose or liquid stools per day because of abnormally high fluid content of stool or an abnormal increase in daily stool frequency^3^.

Fever in children is caused commonly by acute upper respiratory tract infection, and diarrhea in children can be caused by intestinal infection, improper feeding or allergy^1^. Fever and diarrhea are the most common disease symptoms in children, and are key causes of malnutrition, growth and development disorders, and death in children^4^. Moreover, the two-week prevalence of fever and diarrhea is an important indicator for measuring children’s medical and health service needs and reflecting children’s health status. Fever and diarrhea are major public health problems among children under 5 years globally, especially in low- and middle-income countries (LMICs) where the 2-week prevalence rates of fever and diarrhea are 18.8% and 12.5%, respectively^5^. The Sixth National Health Service Statistical Survey Report in 2018 showed the two-week prevalence rates of fever and diarrhea among children under six years in China were 16.1% and 0.9%, respectively, showing significant age and urban–rural differences^6^. The prevalence of fever was similar to that of LMICs, and the prevalence of diarrhea was significantly lower than that of LMICs^5,7^.

Recently, many studies have focused on the prevalence and identification of associated factors of fever and diarrhea in children^8–16^. Factors such as children's age, nutritional status, caregiver's education, family economic status, sanitation, source of drinking water, hand hygiene, and vaccination are closely related to the occurrence of fever and diarrhea in children^8–16^. However, most studies have focused on African and South Asian countries^8–16^, and research on associated factors for fever and diarrhea in Chinese children is very limited^17^. The socioeconomic level, health conditions, and medical services of China are quite different from those in African and Southeast Asian countries^6^. In addition, the high-risk group for fever and diarrhea is children aged 6–23 months^1^. However, previous studies have focused on children under 5 years old, but not on children aged 6–23 months^7–10,18–20^. To the best of the authors’ knowledge, no findings are available for identifying the potential risk factors of fever and diarrhea in light of the most recent Nutrition Improvement Program for Children in Poor Areas (NIPCPA) survey. Therefore, to fulfill this gap, here the survey data of NIPCPA from 2016 to 2023 were used to analyze the two-week prevalence of fever, diarrhea, and coexisting fever and diarrhea among children aged 6–23 months in rural areas of Hunan and explore the associated factors, aiming to provide the basis for taking targeted interventions.

## Subjects and methods

### Study design and population

The Information System of NIPCPA is a national, community-based monitoring system used to monitor and evaluate the nutrition and health status of children in poor rural China where NIPCPA is implemented. NIPCPA implemented by the National Health Commission provides free nutritional supplements rich in proteins, vitamins and minerals (referred to as Yingyangbao) to children aged 6–23 months in poor areas. Moreover, the knowledge and skills of scientific feeding of children are popularized to parents to improve the nutritional status and health level of children in poor areas. Hunan is among the provinces where NIPCPA is implemented. When NIPCPA was launched in 2012 in Hunan, it covered 20 poor counties. It expanded to 25 poor counties in 2014 and further to 51 poor counties in 2018, achieving full coverage of poor counties in the province. As a cross-sectional survey, NIPCPA survey was completed annually in Hunan to collect children’s nutrition and health indicators. The surveyed children were 6 to 23 months old. Each year the survey was administered across 7 counties in Hunan typically from July to October. Based on the nationally unified sampling design^21^, about 2100 children aged 6–23 months from the 7 counties were surveyed each year. We conducted a secondary analysis of the NIPCPA survey data from 2016 to 2023. The exclusion criteria for the surveyed children were: (1) children < 6 months old or ≥ 24 months old; (2) migrant children living in the monitoring area for less than 6 months; (3) children suffering from birth defects, genetic metabolic diseases, endocrine diseases, or immune system diseases.

### Data collection

Monitoring contents included children’s basic information (gender, age, birth weight, gestational age), caregiver status (caregiver type, ethnicity, education level, occupation), feeding status (Yingyangbao consumption, other nutrient supplements, complementary feeding) and illness status (fever, diarrhea) in the past two weeks. The status of complementary feeding was obtained from the caregivers using a past 24-h dietary recall.

### Definition of independent variables

According to the national survey plan for NIPCPA in China, the age of children was divided into three groups: 6–11 months, 12–17 months, and 18–23 months^22^. The birth weight < 2500 g, 2500–3999 g, and ≥ 4000 g were considered as low birth weight, normal birth weight, and macrosomia, respectively^23^. Birth at < 37 gestational weeks was regarded as preterm birth^23^. Caregivers were those who took care of the diets, living and personal security of children, and were divided into two types: parents, and grandparents/others^24^. Ethnicity of caregivers was divided into Han and minorities^25^. The education level of caregivers was classified into primary school and below, junior high school, senior high school, and college or above. The occupation of the caregivers was divided into housework, government agency staff, business service staff, farmers, and others. The Yingyangbao is a complementary food supplement based on soybean powder for infants and young children, and is rich in iron, zinc, calcium, vitamins A, D, B1, B2, B12, folic acid, and other micronutrients^26^. The specific nutrient composition and dosage of Yingyangbao were shown in Table 1. The recommended Yingyangbao intake was one sachet per day. According to the standards of the National Health Commission, children who consume ≥ 4 sachets/week Yingyangbao are considered as effective consumption, and children who consume < 4 sachets/week are considered as non-effective consumption^27^. The consumption of Yingyangbao was divided into three groups: non-consumption, non-effective consumption, and effective consumption. Other nutrients refer to other nutrient supplements besides the Yingyangbao, including vitamin D, vitamin A, calcium, iron, zinc, and DHA^28^. In the past 24-h dietary survey for children, the complementary foods were divided into eight groups: breast milk; grains, roots, tubers and plantains; pulses (beans, peas, lentils), nuts and seeds; dairy products (milk, infant formula, yogurt, cheese); flesh foods (meat, fish, poultry, organ meats); eggs; vitamin-A-rich fruits and vegetables; other fruits and vegetables^29^. The 2021 WHO and UNICEF infant and young child feeding evaluation indicators were used to evaluate the feeding situation of children, including minimum dietary diversity (MDD), minimum meal frequency (MMF) and minimum acceptable diet (MAD)^29^. MDD was defined as consumption of ≥ 5 defined food groups during the previous day. MMF was defined as breastfed infants aged 6–8 months and 9–23 months receiving two or three feedings respectively of solid, semi-solid, or soft foods, or as non-breastfed infants aged 6–23 months receiving four feedings of solid, semi-solid, or soft foods or milk feeds where at least one of the feedings included solid, semisolid, or soft foods. MAD referred to the achievement of both MDD and MMF, with the extra requirement that non-breastfed infants shall have received milk at least twice on the previous day.

**Table 1:** Composition and dosage of Yingyangbao.

Composition of nutrient	Dosage/sachet (12 g)
Energy	220 kJ (52.6 kcal)
Protein	3.0 g
Iron	7.5 mg
Zinc	3 mg
Calcium	200 mg
Vitamin A	250 μg RE
Vitamin D	5.0 μg
Vitamin B_1_	0.50 mg
Vitamin B_2_	0.50 mg
Vitamin B_12_	0.50 mg
Folic acid	75.0 μg

### Definition of outcome variables

The outcome variables were fever, diarrhea, and the coexisting fever and diarrhea among children. The two-week prevalence of fever, diarrhea, and coexisting fever and diarrhea was detected based on the self-report of child caregivers, and refers to the presence of fever and/or diarrhea symptoms in the two weeks preceding the survey. The two-week prevalence rate of fever, diarrhea, or coexisting diseases refers to the ratio of the actual number of patients in the two weeks of survey to the total number of people surveyed^6^. The question “Did your child suffer from fever and/or diarrhea in the past two weeks?” was asked to the caregiver of the child to evaluate the prevalence of illnesses. In NIPCPA survey, fever refers to an axillary temperature exceeding 37 °C, the temperature difference fluctuating by more than 1 °C within 24 h, and reception of advice or treatment from health facilities^22^. Given safety and convenience factors, the temperatures of children were measured using underarm temperature measurement by nurses from health facilities. Specifically, the child's armpit was dried, and place with the mercury column end of the thermometer. Then the child's upper arm was pressed tightly against the armpit, holding for 5–10 min, and the device was taken out for reading. Diarrhea refers to an increase in the frequency of defecation, thin feces, or mucus, pus and blood, and undigested food, such as loose stools, excretion ≥ 3 times a day, or the total daily feces > 200 g, with water content > 80% in the feces, and reception of advice or treatment from health facilities^22^. The coexisting fever and diarrhea refer to the simultaneous presence of both illnesses in a child in the two weeks preceding the survey.

### Statistical analysis

Statistical analysis was conducted on SPSS 25.0. Categorical data were statistically described as proportion or rate. The two-week prevalence of fever, diarrhea, and coexisting fever and diarrhea in children with different characteristics was compared using Chi-square test. With illness status (fever, diarrhea, and coexisting fever and diarrhea) as outcome variables (No = 0, Yes = 1), stepwise logistic regression models (Backward: LR) were developed to identify the associated factors of fever, diarrhea, and coexisting fever and diarrhea in children. To control all possible confounders, all 13 factors related to the characteristics of children, caregivers, and feeding status were included as independent variables in the final model. The results of regression analysis were assessed using OR and 95% CI. The statistical significance was set at *P* < 0.05.

### Ethics approval and consents to participate

The study protocol was approved by the Ethics Committee of Hunan Provincial Maternal and Child Health Care Hospital (No. 2019-S036). The study was conducted in accordance with the *Declaration of Helsinki*. Written informed consents were obtained from all the caregivers of children involved in the annual NIPCPA survey.

## Results

### Characteristics of children

**Figure 1:**
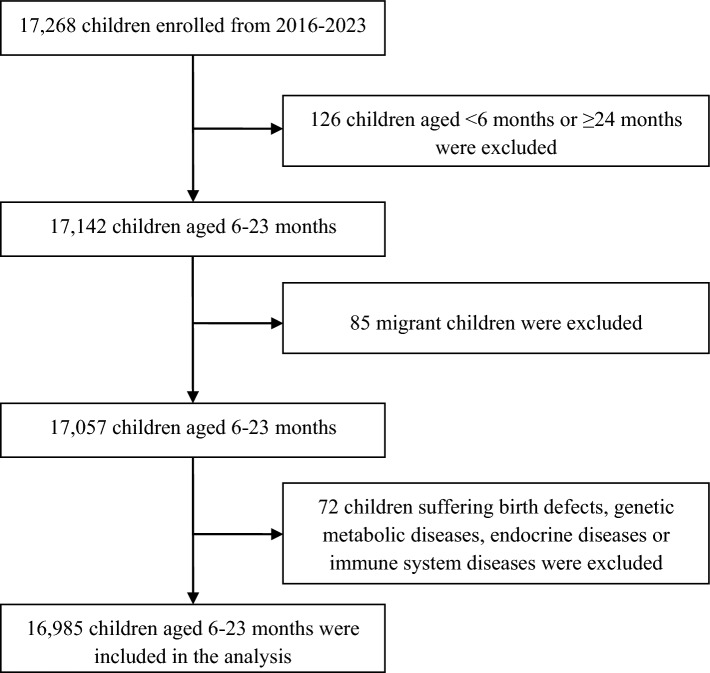
Flowchart of children recruitment.

The recruitment of children is shown in Fig. 1. In total, 17,268 children aged 6–23 months were surveyed from 2016 to 2023. Of them, 126 children aged < 6 months or ≥ 24 months, 85 migrant children, and 72 children suffering birth defects, genetic metabolic diseases, endocrine diseases or immune system diseases were excluded. Finally, 16,985 children involving 51.8% boys were included in the analysis. Children aged 6–11 months, 12–17 months and 18–23 months each accounted for about one-third. The rates of low birth weight and preterm birth were 5.1% and 5.5%, respectively. The caregivers were mostly parents (67.1%). The ethnicity of the caregivers was mostly Han (69.4%), the education level was mainly junior high school (56.1%), and the dominant occupation was housework (49.2%). In terms of children's feeding status, 22.6% of the children did not consume Yingyangbao, 23.9% did not consume Yingyangbao effectively, and 53.5% effectively consumed Yingyangbao; 20.1% of children consumed other nutrient supplements besides Yingyangbao. The proportions that met the MDD, MMF and MAD were 76.8%, 80.3% and 62.5%, respectively (Table 2).

**Table 2:** Univariate analysis of fever, diarrhea, and coexisting fever and diarrhea among children (*N* = 16,985). ^*^P < 0.05.

Characteristics	Frequency (%)	2-week fever				2-week diarrhea				Coexisting fever and diarrhea			
		*n*	%	χ^2^-value	*P*-value	*n*	%	χ^2^-value	*P*-value	*n*	%	χ^2^-value	*P*-value
Characteristics of children													
Gender				7.768	0.005*			3.514	0.061			1.366	0.242
Male	8794 (51.8)	1129	12.8			882	10.0			294	3.3		
Female	8191 (48.2)	937	11.4			752	9.2			248	3.0		
Age group				33.892	< 0.001*			62.860	< 0.001*			57.934	< 0.001*
6–11 months	5826 (34.3)	785	13.5			654	11.2			259	4.4		
12–17 months	5475 (32.2)	705	12.9			575	10.5			172	3.1		
18–23 months	5684 (33.5)	576	10.1			405	7.1			111	2.0		
Birth weight				2.455	0.293			1.928	0.381			0.328	0.849
< 2500 g	868 (5.1)	92	10.6			94	10.8			30	3.5		
2500–3999 g	15,017 (88.4)	1833	12.2			1429	9.5			479	3.2		
≥ 4000 g	1100 (6.5)	141	12.8			111	10.1			33	3.0		
Preterm birth				3.410	0.065			0.593	0.441			0.482	0.488
No	16,057 (94.5)	1971	12.3			1538	9.6			516	3.2		
Yes	928 (5.5)	95	10.2			96	10.3			26	2.8		
Characteristics of caregivers													
Type of caregivers				0.065	0.799			0.243	0.622			0.575	0.448
Parents	11,402 (67.1)	1392	12.2			1088	9.5			372	3.3		
Grandparents/other	5583 (32.9)	674	12.1			546	9.8			170	3.0		
Ethnicity of caregivers				2.972	0.085			3.106	0.078			0.517	0.472
Han ethnicity	11,796 (69.4)	1401	11.9			1166	9.9			384	3.3		
Minorities	5189 (30.6)	665	12.8			468	9.0			158	3.0		
Education level of caregivers				11.253	0.010*			2.651	0.449			1.154	0.764
Primary school or below	1058 (6.2)	150	14.2			106	10.0			36	3.4		
Junior high school	9524 (56.1)	1174	12.3			939	9.9			306	3.2		
Senior high school	4151 (24.4)	509	12.3			390	9.4			136	3.3		
College or above	2252 (13.3)	233	10.3			199	8.8			64	2.8		
Occupation of caregivers				27.041	< 0.001*			7.900	0.095			6.989	0.136
Housework	8357 (49.2)	1112	13.3			851	10.2			296	3.5		
Government agencies staff	1743 (10.3)	163	9.4			153	8.8			50	2.9		
Business services staff	1712 (10.1)	202	11.8			153	8.9			47	2.7		
Farmer	2508 (14.8)	296	11.8			218	8.7			76	3.0		
Other	2665 (15.7)	293	11.0			259	9.7			73	2.7		
Feeding status of children													
Consumption of Yingyangbao				45.993	< 0.001*			53.266	< 0.001*			57.641	< 0.001*
Non-consumption	3835 (22.6)	534	13.9			433	11.3			175	4.6		
Non-effective consumption	4057 (23.9)	570	14.0			466	11.5			162	4.0		
Effective consumption	9093 (53.5)	962	10.6			735	8.1			205	2.3		
Other nutrient supplements				0.247	0.619			0.011	0.916			0.190	0.663
No	13,569 (79.9)	1642	12.1			1307	9.6			437	3.2		
Yes	3416 (20.1)	424	12.4			327	9.6			105	3.1		
Minimum dietary diversity (MDD)				102.524	< 0.001*			54.210	< 0.001*			63.555	< 0.001*
No	3945 (23.2)	662	16.8			499	12.6			203	5.1		
Yes	13,040 (76.8)	1404	10.8			1135	8.7			339	2.6		
Minimum meal frequency (MMF)				10.450	0.001*			0.058	0.809			9.743	0.002*
No	3340 (19.7)	461	13.8			325	9.7			135	4.0		
Yes	13,645 (80.3)	1605	11.8			1309	9.6			407	3.0		
Minimum acceptable diet (MAD)				169.759	< 0.001*			71.487	< 0.001*			44.624	< 0.001*
No	7097 (41.8)	1137	16.0			843	11.9			391	4.0		
Yes	9888 (58.2)	929	9.4			791	8.0			151	2.1		
Total	16,985 (100.0)	2066	12.2			1634	9.6			542	3.2		

### Prevalence of fever, diarrhea, and coexisting fever and diarrhea in children

The two-week prevalence of fever, diarrhea, and coexisting fever and diarrhea among the surveyed children were 12.2% (2066/16,985), 9.6% (1634/16,985), and 3.2% (542/16,985), respectively. Univariate analysis showed that eight factors were related to the occurrence of fever in children, including children’s gender, age, education level of caregivers, occupation, consumption of Yingyangbao, MDD, MMF and MAD. Four factors were related to the occurrence of diarrhea in children, including children's age, consumption of Yingyangbao, MDD and MAD. Five factors were related to the occurrence of coexisting fever and diarrhea in children, including children’s age, consumption of Yingyangbao, MDD, MMF and MAD (Table 2).

### Factors affecting fever, diarrhea, and coexisting fever and diarrhea in children

Table 3 shows the results of multivariate analysis for fever, diarrhea, and coexisting fever and diarrhea in children. Multivariate logistic regression analysis showed that the risk of fever was higher among younger children. Age groups of 6–11 months and 12–17 months were significantly associated with an increased risk of fever, with aORs of 1.20 (95%CI: 1.06–1.35), and 1.32 (1.18–1.49), respectively. The high educational level of caregivers, effective consumption of Yingyangbao, and complementary feeding meeting MDD and meeting MAD were protective factors against fever in children, with aORs of 0.87 (95%CI: 0.78–0.98), 0.78 (0.69–0.87), 0.73 (0.65–0.82), and 0.74 (0.66–0.84), respectively. The risk of diarrhea was higher among younger children. Age groups of 6–11 months and 12–17 months were significantly associated with an increased risk of diarrhea, with aORs of 1.48 (95%CI: 1.29–1.71), and 1.54 (1.34–1.75), respectively. Effective consumption of Yingyangbao, and complementary feeding meeting MDD and MAD were protective factors against diarrhea among children, with aORs of 0.72 (95%CI: 0.63–0.83), 0.79 (0.70–0.91), and 0.80 (0.70–0.92), respectively. The risk of coexisting fever and diarrhea was higher among younger children. Age groups of 6–11 months and 12–17 months were significantly associated with an increased risk of coexisting fever and diarrhea, with aORs of 1.95 (95%CI: 1.55–2.47), and 1.66 (1.30–2.11), respectively. Effective consumption of Yingyangbao, and complementary feeding meeting MDD and MAD were protective factors against coexisting fever and diarrhea among children, with aORs of 0.53 (95%CI: 0.43–0.66), 0.71 (0.58–0.89), and 0.70 (0.56–0.88), respectively.

**Table 3:** Associated factors of fever, diarrhea, and coexisting fever and diarrhea among children in multivariate analysis.

Factors	2-week fever			2-week diarrhea			Coexisting fever and diarrhea		
	*aOR*	95%*CI*	*P*-value	*aOR*	95%*CI*	*P*-value	*aOR*	95%*CI*	*P*-value
Age group									
6–11 months	1.20	1.06–1.35	0.002*	1.48	1.29–1.71	< 0.001*	1.95	1.55–2.47	< 0.001*
12–17 months	1.32	1.18–1.49	< 0.001*	1.53	1.34–1.75	< 0.001*	1.66	1.30–2.11	< 0.001*
18–23 months	1.00			1.00			1.00		
Education level of caregivers	0.87	0.78–0.98	0.008*						
Consumption of Yingyangbao									
Non-consumption	1.00			1.00			1.00		
Non-effective consumption	1.01	0.89–1.15	0.823	1.06	0.92–1.20	0.600	0.90	0.72–1.12	0.350
Effective consumption	0.78	0.69–0.87	< 0.001*	0.72	0.63–0.83	< 0.001*	0.53	0.43–0.66	< 0.001*
Minimum dietary diversity	0.73	0.65–0.82	< 0.001*	0.79	0.70–0.91	0.001*	0.71	0.58–0.89	0.002*
Minimum acceptable diet	0.74	0.66–0.84	< 0.001*	0.80	0.70–0.92	0.001*	0.70	0.56–0.88	0.003*

^a^Analyses were adjusted for children’s gender, age, birth weight, preterm birth, caregivers’ type, ethnicity, educational level, occupation, consumption of Yingyangbao, other nutrient supplements, minimum dietary diversity, minimum meal frequency, and minimum acceptable diet. *aOR* denotes adjusted odds ratio. *CI* denotes confidence interval. *P < 0.05.

## Discussion

The 2-week prevalence of fever and diarrhea in children can reflect their health status and medical and health service needs. Using the information provided by caregivers of 16,985 children aged 6–23 months from NIPCPA survey data, we explored the associated factors of fever, diarrhea, and coexisting fever and diarrhea in rural areas of Hunan. The 2-week prevalence rates of fever, diarrhea, and coexisting fever and diarrhea among children were 12.2%, 9.6%, and 3.2%, respectively. Our major findings are that age of children, educational level of caregivers, effective consumption of Yingyangbao, complementary feeding meeting MDD and MAD are associated with fever, and age of children, effective consumption of Yingyangbao, complementary feeding meeting MDD and MAD are associated with diarrhea, and coexisting fever and diarrhea.

The 2-week prevalence of fever in this study was lower than that of children aged ≤ 2 years in rural areas across China in 2018 (17.9%), and the 2-week prevalence of diarrhea was higher than that of children aged ≤ 2 years in rural areas across China in 2018 (1.4%)^6^. The 2-week prevalence of fever and diarrhea was significantly lower than the levels of children under 5 years old in African and South Asian countries (18.8–34.0% for fever, and 12.0–29.9% for diarrhea)^5,8,12,15,18,19^, and the two-week prevalence of coexisting fever and diarrhea was similar to that in Bangladesh (3.0%)^8^. Previous studies show that factors such as socioeconomic status, sanitation conditions, and safe drinking water are closely related to the occurrence of fever and diarrhea in children^9,11,12^. The socioeconomic level, sanitation facilities and safe drinking water of China are significantly better than those in African and South Asian countries^6^, resulting in a lower two-week prevalence of fever and diarrhea in children compared to these countries.

The present study showed the 2-week prevalence of fever, diarrhea, and coexisting fever and diarrhea was higher among younger children. The two-week risks of fever among children aged 6–11 months and 12–17 months were 1.20 and 1.32 times higher respectively, the 2-week risks of diarrhea were 1.48 and 1.53 times higher respectively, and the two-week risks of coexisting fever and diarrhea were 1.95 and 1.66 times higher respectively than in children aged 18–23 months. The possible reason was that the respiratory system, digestive system and immune system were not yet mature in these young children (except the age within 6 months), and the fetal-transmitted antibodies from the mothers during the fetal periods had basically subsided, resulting in low immunity and proneness to respiratory and respiratory tract infections that caused fever and diarrhea. This finding is consistent with previous studies that children aged 6–11 months and 12–23 months had the highest risks of fever and diarrhea among children under 5 years of age^10,30^.

Previous studies confirm that caregivers’ education level is associated with fever and diarrhea in children, and a lower educational level of caregivers leads to a higher risk of developing fever and diarrhea in children^18,30^. A study conducted in sub-Saharan African countries showed children of mothers with no education or primary education were 1.69 and 1.87 times, respectively, more likely to suffer from diarrhea than children of mothers with higher education^18^. The present study revealed that a high education level of caregivers was a protective factor for fever in children, but it has not been found that the education level of caregivers was related to diarrhea, or coexisting fever and diarrhea in children. Although no relationship was found between caregivers’ education level and childhood diarrhea, coexisting fever and diarrhea in the multivariate analysis of this study, the two-week prevalence of diarrhea, and coexisting fever and diarrhea in children also mildly decreased with the improvement of caregivers’ education level in the univariate analysis, which was not significant due to the small decrease. This is not surprising given that education enables mothers to be well informed about how to find and utilize appropriate child health information^20^.

Moreover, effective consumption of Yingyangbao was a protective factor for fever, diarrhea, and coexisting fever and diarrhea in children, with the risks of 0.78, 0.72 and 0.53 times, respectively, lower than in children not consuming Yingyangbao, which is consistent with the results in Henan, China^17^. A cross-sectional study from Henan showed that effective consumption of Yingyangbao could reduce the two-week prevalence of fever and diarrhea in children^17^. This is because the Yingyangbao contains vitamin A, zinc and other nutrients. Vitamin A and zinc have a wide range of biological effects and can maintain and promote immune functions^1^. The most common causes of fever or diarrhea in children are upper respiratory tract or intestinal infections. Some studies confirm that vitamin A and zinc can reduce the risk of infection from pathogenic vitamins in children and shorten the course of diseases, such as upper respiratory tract infections and infectious diarrhea^31,32^. In addition, the Yingyangbao contains a reasonable amount of iron, which avoids side effects such as diarrhea, vomiting, and fever caused by high doses of iron^26^.

For children, the age of 6–23 months is a critical period for growth and development. Scientific and reasonable complementary feeding during this period is the basis for children's good nutritional status. WHO recommends that infants continue breastfeeding and start complementary foods when they are 6 months old^33^. Adding complementary foods too early, too late, or unreasonably will have adverse effects on children's health. A multicenter cross-sectional study conducted in Ethiopia showed that starting complementary feeding before 6 months of age increased the risk of acute diarrhea in children to 6.49 times^9^. Our study found that feeding complementary foods to meet MDD and MAD would reduce the risk of fever in children by 27% and 26%, respectively, the risk of diarrhea by 21% and 20%, respectively, and the risk of coexisting fever and diarrhea by 29% and 30%, respectively. Scientific and reasonable complementary feeding would provide good nutritional support for the growth and development of children, avoiding malnutrition and improving body immunity^34,35^.

This study has some limitations. First, this was a cross-sectional study conducted in different years. Owing to the inherent characteristics of cross-sectional studies, the relationship between investigated factors and children’s fever or diarrhea identified in our study was a statistical association, rather than causality. Second, the collection of children’s fever and diarrhea data was based on caregivers’ self-reported results, which largely relied on the caregivers’ cognitive ability of children's health and disease conditions, and inevitably contained information bias. Third, due to limitations in data collection, some drinking-water, sanitation and hygiene (WASH) factors related to fever and diarrhea (e.g., drinking-water source, sanitation facilities, hand washing, food hygiene) were unavailable in our study. Therefore, these factors were not included in the final analysis, which might lead to a certain degree of overestimation of the effects of investigated factors on children’s fever and diarrhea. In future studies, we will comprehensively and systematically collect data, including WASH factors and medical services conditions, to further explore the key determinants of fever and diarrhea in children and lay a solid foundation for future intervention research on children’s fever and diarrhea.

## Conclusions

In rural areas of Hunan, the 2-week prevalence of fever, diarrhea, and coexisting fever and diarrhea among children aged 6–23 moths can be expressed as follows: one in every eight children suffers from fever, one in every ten children suffers from diarrhea, and one in every thirty children suffers from coexisting fever and diarrhea. Age of children, education level of caregivers, consumption of Yingyangbao, and complementary feeding were identified as associated factors for childhood illness. Effective interventions should be actively taken, such as improving the education level of caregivers, enhancing their scientific feeding skills for children, and promoting the children’s compliance with Yingyangbao consumption, to further reduce the prevalence of fever and diarrhea in children.

## Data Availability

The datasets generated during and/or analysed during the present study are not publicly available due to the personal privacy of subjects, but are available from the corresponding author on reasonable request.
